# Idiopathic Peroneal Tenosynovitis Caused by a Previously Undiagnosed Auto-immune Condition

**DOI:** 10.7759/cureus.2229

**Published:** 2018-02-26

**Authors:** Pejma Shazadeh Safavi, Cory F Janney, Kenrick Lam, Daniel R Kunzler, Vinod Panchbhavi

**Affiliations:** 1 University of Texas Medical Branch at Galveston; 2 Department of Orthopedics, United States Navy, University of Texas Medical Branch at Galveston; 3 Orthopedics, University of Texas Medical Branch at Galveston; 4 School of Medicine, University of Texas Medical Branch at Galveston; 5 Department of Orthopedics, University of Texas Medical Branch at Galveston

**Keywords:** peroneal, tenosynovitis, idiopathic, autoimmune

## Abstract

In this case report, we present a unique case of idiopathic peroneal tenosynovitis in an otherwise healthy patient, presenting with a three-month history of pain over the lateral aspect of the right foot. Imaging revealed that fluid distention and synovial thickening distend the common peroneal tendon sheath and peroneus longus and brevis tendon sheaths. The patient was managed operatively with excision of the peroneus longus tendon, a side-to-side tenodesis, and Bröstrom-Gould lateral ankle ligament repair. Histologic examination was suggestive of a chronic inflammatory process possibly due to underlying autoimmune etiology. At three-month follow-up, the patient reported complete resolution of pain and is resuming normal activities without difficulty.

## Introduction

The etiology of peroneal tenosynovitis stems from chronic or acute mechanical irritation, infections, and prior rheumatologic disorders. Histology often shows evidence of chronic inflammation [1-4]. We present a case of isolated inflammatory histology with germinal centers in the common peroneal tendon, confirmed by pathologic examination of several specimens following surgical intervention. The patient presented in this case showed no other articular or extra-articular signs of rheumatoid arthritis and presented initially for evaluation of chronic ankle pain.

## Case presentation

**Figure 1:**
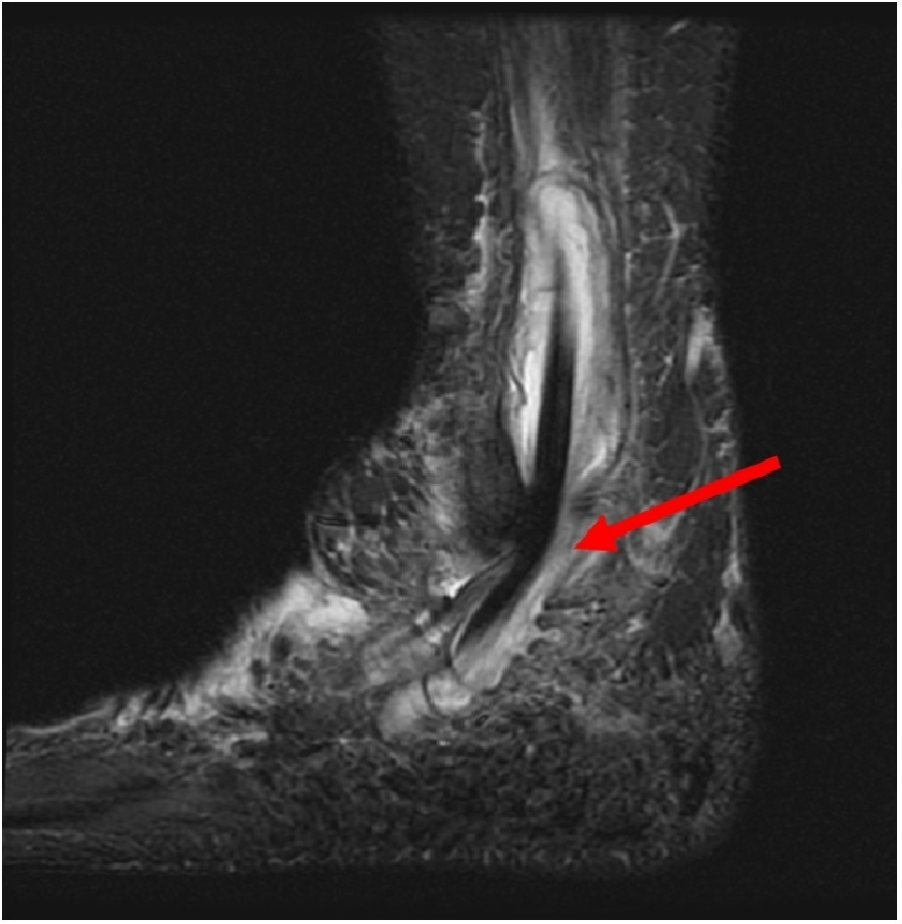
Lateral 3T MRI of the right foot. Marked fluid distention and synovial thickening distend the common peroneal tendon sheath and peroneal tendons (arrow). MRI: Magnetic resonance imaging.

**Figure 2:**
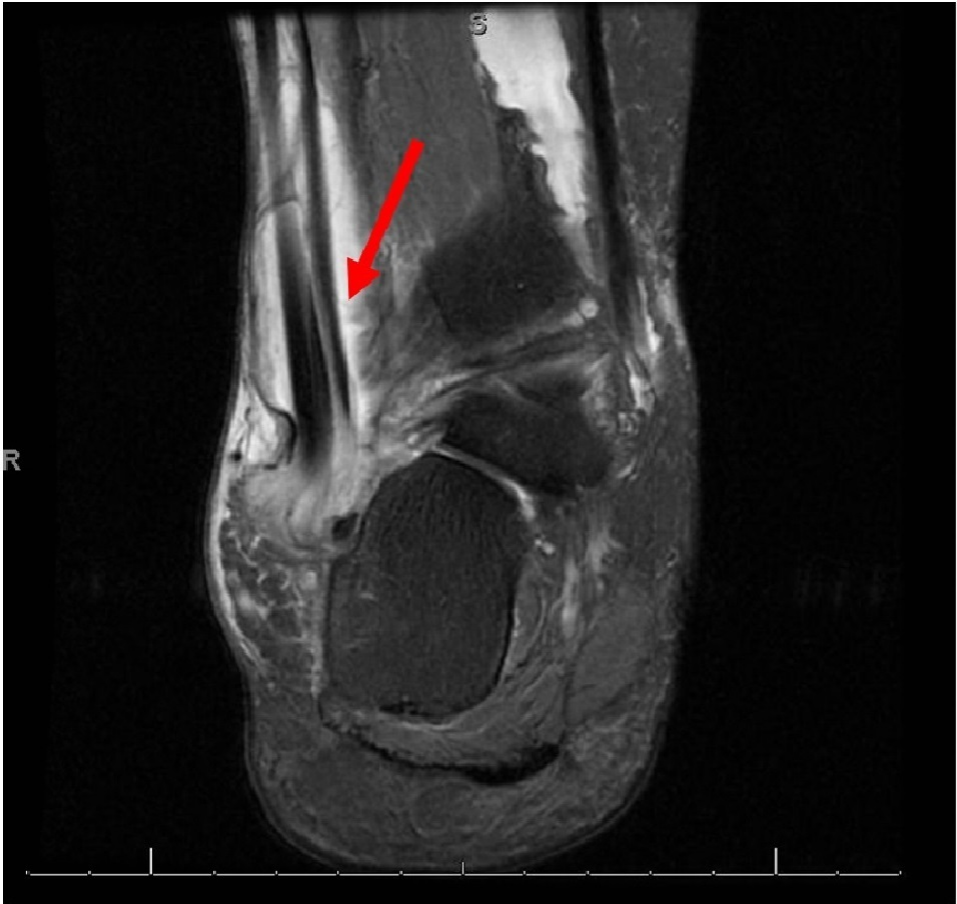
Coronal 3T MRI of the right foot. Severe peroneal tenosynovitis with interstitial peroneal tendon tearing (arrow). MRI: Magnetic resonance imaging.

A 53-year-old female presented to clinic with pain over the lateral aspect of the right foot which began after ‘rolling’ her right ankle three months prior. She reported that two previous attempts of conservative management with a fracture boot for six weeks, which was subsequently repeated for three weeks, did not improve her pain. Multi-planar, multi-weighted, 3 Tesla (3T) magnetic resonance imaging (MRI) of the right ankle revealed the following conditions: 1) chondral thinning over the medial margin of the tibial-talar joint, with underlying bone marrow edema at the level of the medial talar dome, 2) marked fluid distension and synovial thickening with distension of the common peroneal tendon sheath into the peroneus brevis and peroneus longus sheaths, and 3) reactive subcortical bone marrow edema along the lateral wall of the calcaneus. The peroneal tendons demonstrated tearing and degenerative flattening (Figure 1 and Figure 2). Inflammatory changes were noted along the posterior tibialis tendon and flexor digitorum longus. Given these findings, the initial assessment was a chronic ankle sprain with peroneal tenosynovitis.

The patient was taken to the operating room for exploration and planned tendon debridement and repair. A lateral ankle incision was placed along the fibula to allow access to both the attenuated anterior talofibular ligament (ATFL) area as well as the retrofibular space to evaluate the peroneal tendons. Careful dissection was performed after elevating skin flaps to avoid damage to neurovascular structures. The superior peroneal retinaculum was incised, and a copious amount of yellow fluid egressed from the distended peroneal tendon sheath. Upon entering the peroneal tendon sheaths, a significant amount of brownish-gray synovial proliferation was noted throughout the retrofibular space. Several samples were taken from the peroneal tendon sheath synovium, peroneus brevis, and peroneus longus, and sent to pathology to determine the cause of this proliferation. The peroneus longus tendon was noted to be torn, with >80% of its length disrupted and degenerated. At that time, a decision was made to excise the peroneus longus tendon and perform a side-to-side tenodesis. A Broström-Gould lateral ankle ligament repair was also performed.

At first follow-up, nine days postoperatively, the patient’s lateral ankle wound was healed and her pain was improving. She was referred for physical therapy but was unable to make the appointment due to personal issues. At three months postoperatively, the patient was called as she had missed her second follow-up due to continued personal issues. She declared that her pain prior to surgery had resolved completely and she was back to all of her activities without difficulty.

**Figure 3:**
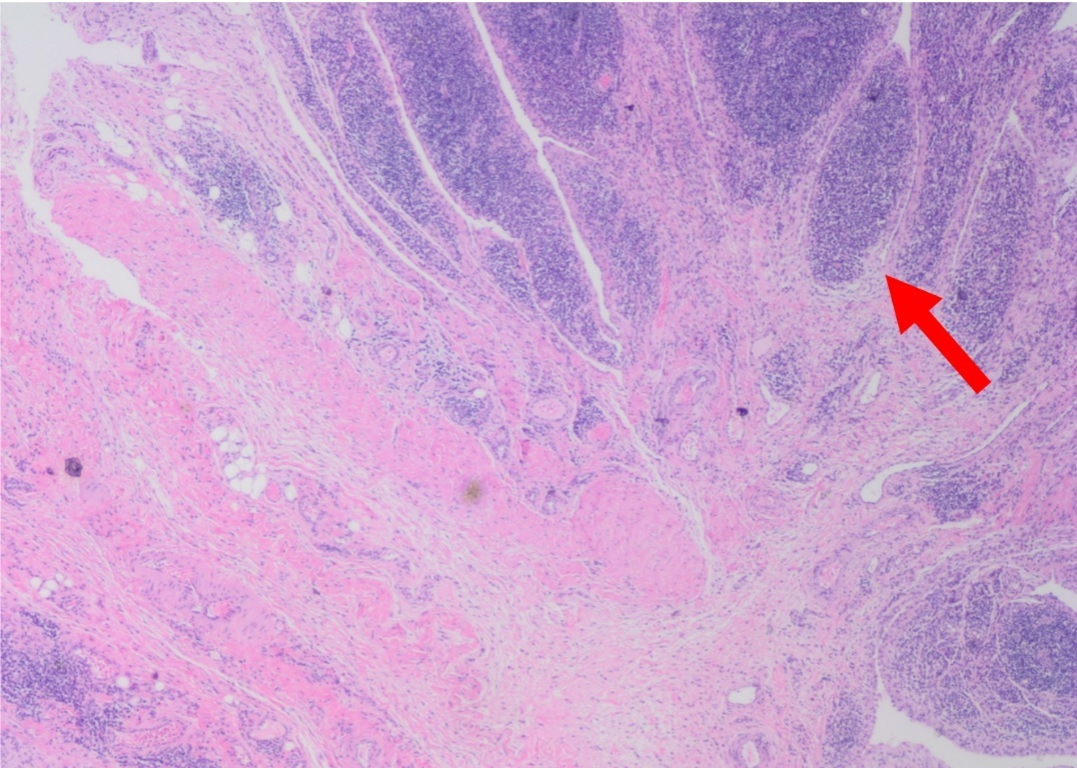
Right peroneal tendon mass, excision. Fibroadipose tissue with marked lymphoplasmacytic infiltration. The synovium contains dense acute and chronic inflammation with germinal centers (arrow).

**Figure 4:**
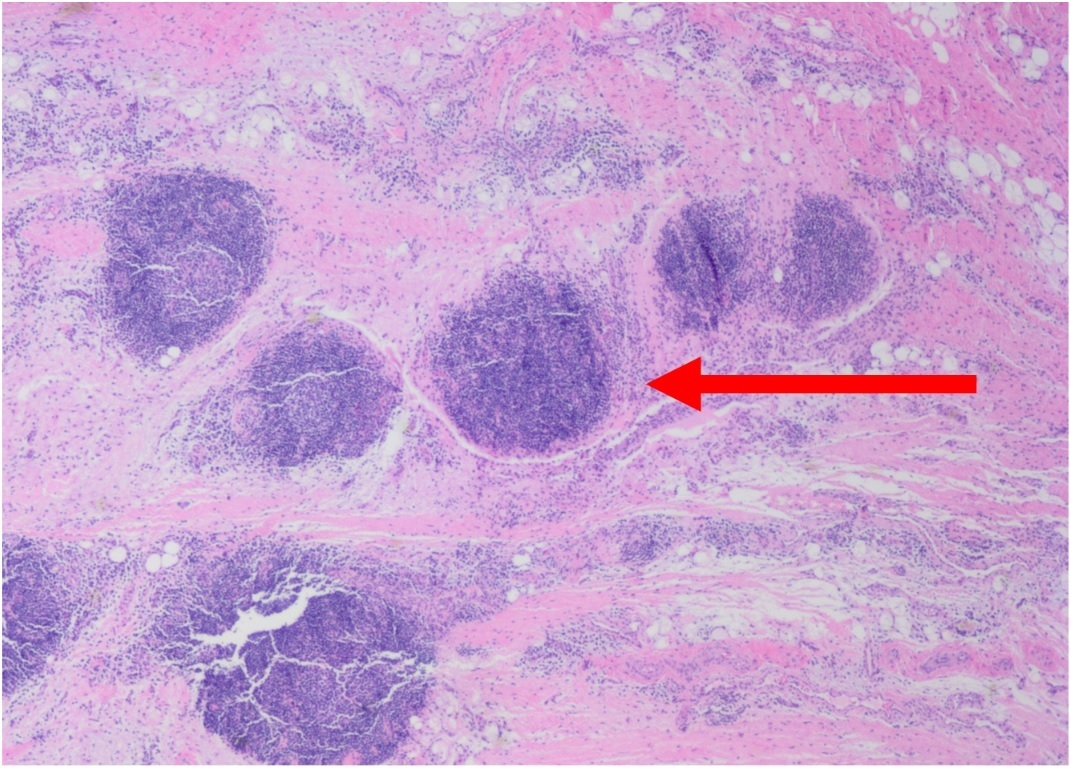
Right peroneus brevis, excision. Fibroadipose tissue with marked lymphoplasmacytic infiltration. The synovium contains dense acute and chronic inflammation with germinal centers (arrow).

**Figure 5:**
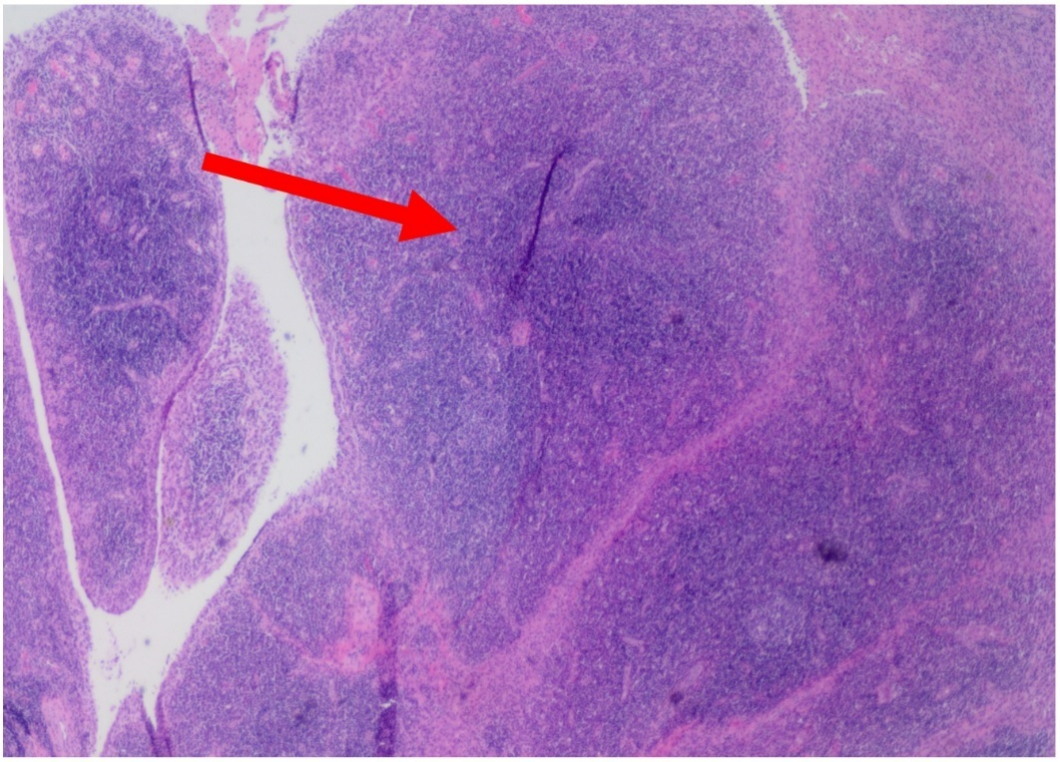
Right peroneus longus tendon, excision. Fibroadipose tissue with marked lymphoplasmacytic infiltration. The synovium contains dense acute and chronic inflammation with germinal centers (arrow).

Specimens from the peroneus brevis, peroneus longus, and peroneal tendon sheath synovium were evaluated by pathology. Tissue from all tendons demonstrated marked lymphocytic inflammation with chronic and acute inflammation and development of germinal centers observed within the synovium (Figures 3-5).

## Discussion

Peroneal tendonitis often stems from traumatic etiology or in the presence of predisposing anatomy, such as in patients with a large peroneal tubercle or low-lying muscle belly. In these situations, typical histology of uncomplicated tenosynovitis is progressive and involves three distinct grades. Grades I–III correspond to isolated synovial fibrous hypertrophy of the synovium, with later development of localized necrotic areas, and progressive development of empty spaces reminiscent to serous bursae, respectively [1-3].

When history and physical examination do not point to a mechanical etiology for a patient’s pain, infectious or rheumatologic etiologies should be considered. Even then, the etiology of the tenosynovitis may be difficult to ascertain. Our case demonstrates the potential value of sending the excised portions of tenosynovium for histology during operative treatment of idiopathic peroneal tenosynovitis. In this case, the dense lymphocytic infiltrate found was suggestive of possible underlying rheumatologic disease [1]. Although rare, tenosynovitis has been described as the presenting complaint of patients who eventually were diagnosed with rheumatoid arthritis [4]. The pathology appears similar to that of rheumatoid arthritis; however, without formal joint involvement, this specific diagnosis cannot be made. This case suggests that auto-immunity may in fact be an underlying cause of irritation in patients with otherwise idiopathic peroneal tenosynovitis.

## Conclusions

Histology of this patient’s idiopathic peroneal tenosynovitis was suggestive of undiagnosed autoimmune disease. Peroneal tenosynovitis has not been documented as an initial finding in undiagnosed immune disease, but similar histologic changes observed in some cases of carpal tunnel syndrome have been noted as initial signs of autoimmune disease. Surgical treatment of idiopathic tenosynovitis is an appropriate treatment choice due to the risk of rupture and progressive deterioration if left untreated.
